# A noncompeting pair of human neutralizing antibodies block COVID-19 virus binding to its receptor ACE2

**DOI:** 10.1126/science.abc2241

**Published:** 2020-05-13

**Authors:** Yan Wu, Feiran Wang, Chenguang Shen, Weiyu Peng, Delin Li, Cheng Zhao, Zhaohui Li, Shihua Li, Yuhai Bi, Yang Yang, Yuhuan Gong, Haixia Xiao, Zheng Fan, Shuguang Tan, Guizhen Wu, Wenjie Tan, Xuancheng Lu, Changfa Fan, Qihui Wang, Yingxia Liu, Chen Zhang, Jianxun Qi, George Fu Gao, Feng Gao, Lei Liu

**Affiliations:** 1 Department of Pathogen Microbiology, School of Basic Medical Sciences, Capital Medical University, Beijing, China.; 2 Research Network of Immunity and Health (RNIH), Beijing Institutes of Life Science, Chinese Academy of Sciences, Beijing, China.; 3 CAS Key Laboratory of Pathogenic Microbiology and Immunology, Institute of Microbiology, Chinese Academy of Sciences (CAS), Beijing, China.; 4 School of Life Sciences, University of Science and Technology of China, Hefei, Anhui, China.; 5 Shenzhen Key Laboratory of Pathogen and Immunity, Shenzhen Third People’s Hospital, Shenzhen, China.; 6 College of Veterinary Medicine, China Agricultural University, Beijing, China.; 7 Laboratory of Protein Engineering and Vaccines, Tianjin Institute of Industrial Biotechnology, Chinese Academy of Sciences (CAS), Tianjin, China.; 8 Shanxi Academy of Advanced Research and Innovation, Taiyuan, China.; 9 University of Chinese Academy of Sciences, Beijing, China.; 10 Center for Influenza Research and Early Warning, Chinese Academy of Sciences (CASCIRE), Beijing, China.; 11 NHC Key Laboratory of Biosafety, National Institute for Viral Disease Control and Prevention, Chinese Center for Disease Control and Prevention, Beijing, China.; 12 Laboratory Animal Center, Chinese Center for Disease Control and Prevention, Beijing, China.; 13 Division of Animal Model Research, Institute for Laboratory Animal Resources, National Institutes for Food and Drug Control, Beijing, China.

## Abstract

Neutralizing antibodies could potentially be used as antivirals against the coronavirus disease 2019 (COVID-19) pandemic. Here, we report isolation of four human-origin monoclonal antibodies from a convalescent patient, all of which display neutralization abilities. The antibodies B38 and H4 block binding between the spike glycoprotein receptor binding domain (RBD) of the virus and the cellular receptor angiotensin-converting enzyme 2 (ACE2). A competition assay indicated different epitopes on the RBD for these two antibodies, making them a potentially promising virus-targeting monoclonal antibody pair for avoiding immune escape in future clinical applications. Moreover, a therapeutic study in a mouse model validated that these antibodies can reduce virus titers in infected lungs. The RBD-B38 complex structure revealed that most residues on the epitope overlap with the RBD-ACE2 binding interface, explaining the blocking effect and neutralizing capacity. Our results highlight the promise of antibody-based therapeutics and provide a structural basis for rational vaccine design.

## An antibody defense against COVID-19

One of the responses of the immune system to invading viruses is the production of antibodies. Some of these are neutralizing, meaning that they prevent the virus from being infectious, and can thus be used to treat viral diseases. Wu *et al.* isolated four neutralizing antibodies from a convalescent coronavirus disease 2019 (COVID-19) patient. Two of the antibodies, B38 and H4, blocked the receptor binding domain (RBD) of the viral spike protein from binding to the cellular receptor, angiotensin-converting enzyme 2 (ACE2). The structure of the RBD bound to B38 shows that the B38-binding site overlaps with the binding site for ACE2. Although H4 also blocks RBD binding to ACE2, it binds at a different site, and thus the two antibodies can bind simultaneously. This pair of antibodies could potentially be used together in clinical applications.

*Science*, this issue p. 1274

## Teaser

A pair of neutralizing antibodies against COVID-19 bind to different epitopes to compete with cellular receptor binding.

Coronavirus disease 2019 (COVID-19) caused by the novel COVID-19 virus has become a pandemic. The virus has spread worldwide, causing fever, severe respiratory illness, and pneumonia (*1*, *2*). Phylogenetic analysis indicates that the virus is closely related to severe acute respiratory syndrome coronavirus (SARS-CoV) (*3*–*5*), but it appears to be more easily transmitted from person to person than SARS-CoV (*6*). To date, no specific drugs or vaccines are available for COVID-19.

The COVID-19 virus belongs to the betacoronavirus genus, which includes five pathogens that infect humans (*7*, *8*). Among them, SARS-CoV and Middle East respiratory syndrome coronavirus (MERS-CoV) are two highly pathogenic viruses. As with other coronaviruses, the spike (S) glycoprotein homotrimer on the COVID-19 virus surface plays an essential role in receptor binding and virus entry. The S protein is a class I fusion protein—each S protomer consists of S1 and S2 domains (*9*), with the receptor binding domain (RBD) located within the S1 domain (*8*). Previous studies have revealed that the COVID-19 virus, similarly to SARS-CoV, uses the angiotensin-converting enzyme 2 (ACE2) receptor for cell entry (*3*, *10*–*13*). Numerous neutralizing antibodies have been found to target the RBDs of SARS-CoV or MERS-CoV (*14*–*16*). Therefore, screening for neutralizing antibodies that target the COVID-19 virus RBD is a priority.

We expressed COVID-19 virus RBD protein as bait to isolate specific single memory B cells from COVID-19 patient peripheral blood mononuclear cells (PBMCs). The variable regions encoding the heavy and light chains were each amplified from separate single B cells and then cloned into a pCAGGS vector with the constant region to produce immunoglobulin G1 (IgG1) antibodies, as described previously (*17*). Seventeen paired B cell clones were amplified, three of which were identical (B5, B59, and H1). To identify the antibody binding abilities, the plasmids containing the paired heavy and light chains were cotransfected into human embryonic kidney–293T (HEK 293T) cells for monoclonal antibody (mAb) production. The supernatants were then screened for binding to the RBD by biolayer interferometry (BLI). An irrelevant anti–severe fever with thrombocytopenia syndrome virus Gn antibody and a SARS-specific antibody were used as controls (*18*). The supernatants from four different antibodies (B5, B38, H2, and H4) bound to COVID-19 virus RBD but not to SARS-CoV RBD (fig. S1), suggesting that the epitopes of the two RBDs are immunologically distinct. The usage of heavy chain (V_H_) and light chain (V_L_) variable genes in these four antibodies is listed in table S1.

**Fig. 1:**
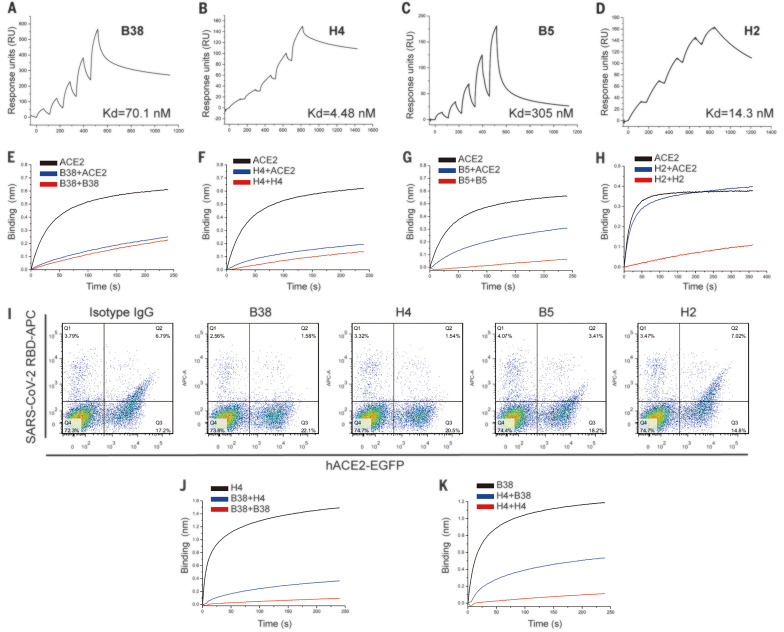
Characterization of COVID-19 virus–specific neutralizing antibodies. (**A** to **D**) The binding kinetics between four antibodies (B38, H4, B5, and H2) and COVID-19 virus RBD were measured using a single-cycle Biacore 8K system. (**E** to **H**) Competition binding to the COVID-19 virus RBD between antibodies and ACE2 was measured by BLI. Immobilized biotinylated COVID-19 virus RBD (10 μg/ml) was saturated with antibodies and then flowed with corresponding antibody in the presence of 300 nM soluble ACE2 (blue) or without ACE2 (red). As a control, the immobilized biotinylated RBD was flowed with buffer and then flowed with the equal molar concentration of ACE2 (black). The graphs show binding patterns after antibody saturation. (**I**) hACE2–enhanced green fluorescent protein (EGFP) was expressed on the HEK293T cell surface, and the cells were stained with 200 ng/ml COVID-19 virus RBD his-tag proteins preincubated with isotype IgG, B38, H4, B5, or H2. The percentages of anti-his-tag APC^+^ (allophycocyanin) cells and EGFP^+^ cells were calculated. (**J** and **K**) Competition binding to COVID-19 virus RBD between B38 and H4 was measured by BLI. Immobilized COVID-19 virus RBD (10 μg/ml) was saturated with 300 nM of the first antibody and then flowed with equal molar concentration of the first antibody in the presence of (blue) or without (red) the second antibody. Equal molar concentration of the second antibody was flowed on the immobilized RBD as a control (black). The graphs show binding patterns after saturation of the first antibody.

**Fig. 2:**
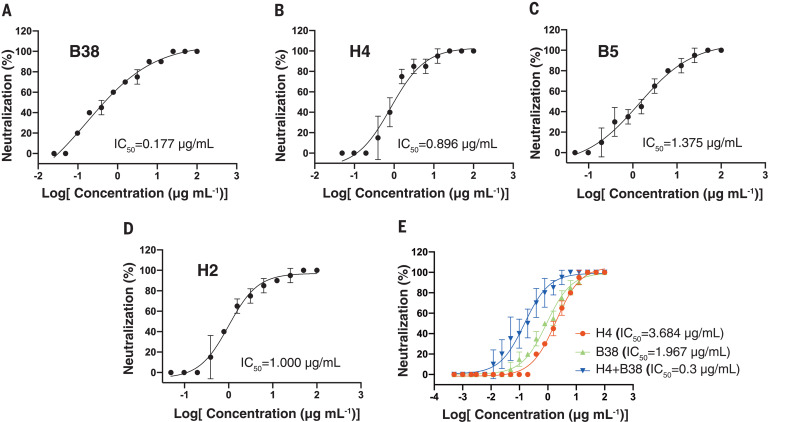
Four antibodies can effectively neutralize COVID-19 virus, and two of them exhibit additive inhibition effect. The mixtures of COVID-19 virus and serially diluted antibodies were added to Vero E6 cells. After 5 days of incubation, IC_50_ values were calculated by fitting the cytopathic effect from serially diluted antibody to a sigmoidal dose-response curve. Medium containing 100 and 200 times the median tissue culture infectious dose of COVID-19 virus was used for testing the neutralizing abilities of individual antibody (**A** to **D**) and cocktail antibodies (**E**), respectively.

The dissociation constants (*K*_d_) for the four antibodies binding to COVID-19 virus RBD, measured using surface plasmon resonance (SPR), ranged from 10^−7^ to 10^−9^ M (Fig. 1, A to D). We next studied the neutralizing activities of these four antibodies against COVID-19 virus (the BetaCoV/Shenzhen/SZTH-003/2020 strain). All four antibodies exhibited neutralizing activities, with median inhibitory concentration (IC_50_) values ranging from 0.177 to 1.375 μg/ml (Fig. 2, A to D). A cocktail of B38 and H4 exhibited synergetic neutralizing ability, even in the presence of a higher virus titer (Fig. 2E).

To evaluate the ability of each antibody to inhibit binding between RBD and ACE2, we performed a competition assay using BLI and a blocking assay using fluorescence-activated cell sorting (FACS). For the BLI assay, streptavidin biosensors labeled with biotinylated RBD were saturated with antibodies, and then the test antibodies were flowed through in the presence of soluble ACE2. B38 and H4 showed complete competition with ACE2 for binding to RBD. In contrast, B5 displayed partial competition, whereas H2 did not compete with ACE2 for RBD binding (Fig. 1, E to H). The blocking assay by FACS presented the same result (Fig. 1I). To determine whether B38 and H4 target the same epitope, we performed an epitope competition assay by BLI. The nickel–nitrilotriacetic acid sensor labeled with the RBD was saturated with B38 IgG, and H4 IgG was flowed through, or the reverse (sensor saturated with H4 IgG, and B38 IgG flowed through). Although RBD was saturated with the first antibody, the second antibody could still bind to RBD, but with some inhibition. This suggests that B38 and H4 recognize different epitopes on RBD with partial overlap (Fig. 1, J and K).

**Fig. 3:**
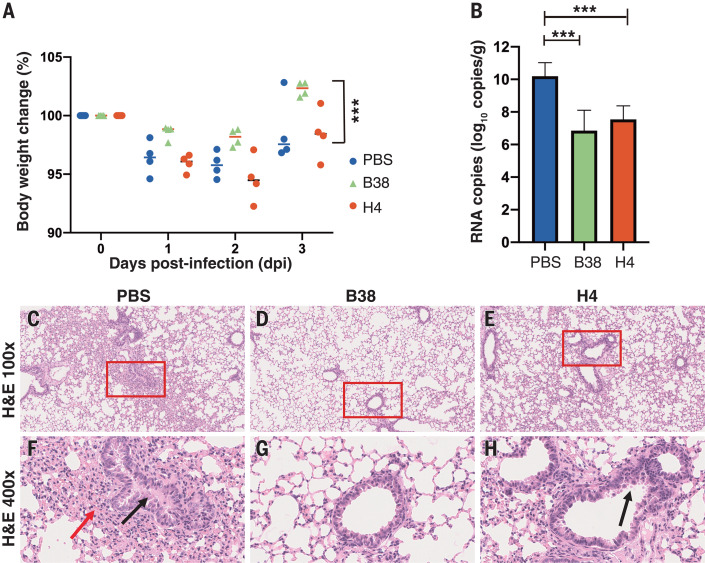
The protection efficiency of mAbs in hACE2 mice model after infection with COVID-19 virus. (**A**) Body weight loss was recorded for PBS, B38 treatment, and H4 treatment groups (for all groups, *n* = 4 mice). All the mice were challenged intranasally with COVID-19 virus, and a 25 mg/kg dose of antibodies was injected (intraperitoneally) 12 hours after infection. Equal volume of PBS was used as a control. The weight loss was recorded over 3 days, and a significant difference could be observed between the B38 group and the PBS group (unpaired *t* test, ***P < 0.001). (**B**) The virus titer in lungs of three groups was determined at 3 dpi by real-time quantitative reverse transcription polymerase chain reaction (qRT-PCR). The mAb treatment group reduced the viral load in the lungs of mice (unpaired *t* test, ***P < 0.001). (**C** to **H**) Representative histopathology of the lungs in COVID-19 virus–infected hACE2 mice (3 dpi). Severe bronchopneumonia and interstitial pneumonia was observed in the PBS group [(C) and (F)], with edema and bronchial epithelial cell desquamation (black arrow) and infiltration of lymphocytes within alveolar spaces (red arrow). Mild bronchopneumonia was observed in the H4 group [(E) and (H)], whereas no lesions were observed in the B38 group [(D) and (G)]. The images and areas of interest (red boxes) are magnified 100× and 400×, respectively.

To explore the protection efficacy of B38 and H4 against challenge with COVID-19 virus in vivo, hACE2 transgenic mice were administered a single 25 mg/kg dose of B38 or H4 12 hours after viral challenge. The body weight of the B38 group decreased slowly and recovered at 3 days postinfection (dpi) compared with the phosphate-buffered saline (PBS) control group and the H4 group (Fig. 3A). The number of viral RNA copies in the lung were also measured at 3 dpi. The RNA copies of both the B38 group and the H4 group were significantly lower than those of the PBS group, with a reduction of 3.347 and 2.655 logs, respectively (Fig. 3B). These results show the same trends as the neutralization abilities. Histopathological examination indicated that severe bronchopneumonia and interstitial pneumonia could be observed in the mice of the PBS control group, with edema and bronchial epithelial cell desquamation and infiltration of lymphocytes within alveolar spaces (Fig. 3, C and F). Mild bronchopneumonia was observed in the H4 group (Fig. 3, E and H), whereas no lesions were observed in the B38 group (Fig. 3, D and G).

**Fig. 4:**
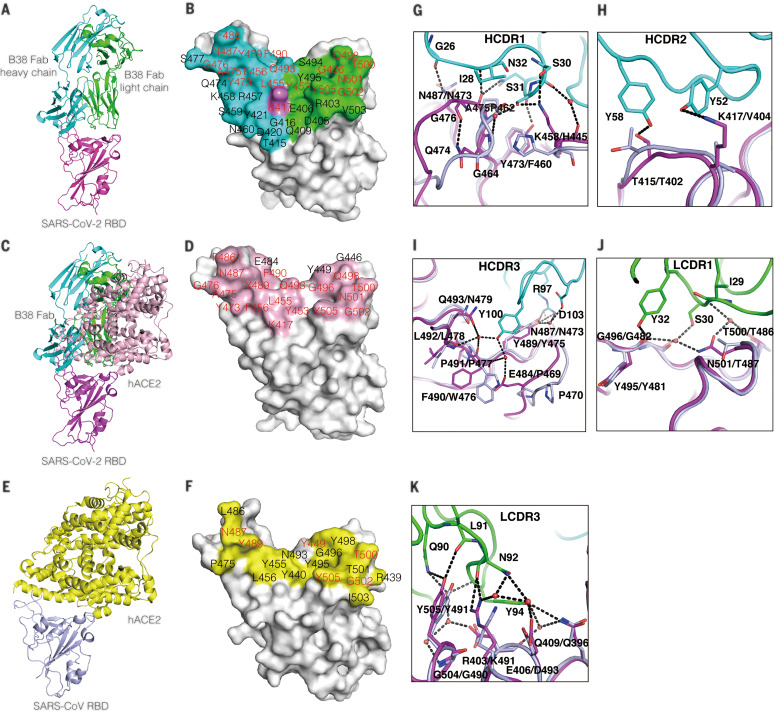
Structural analysis of B38 and COVID-19 virus RBD complex and the epitope comparison between B38 and hACE2. (**A**) The overall structure of B38 Fab and COVID-19 virus RBD. The B38 heavy chain (cyan), light chain (green), and COVID-19 virus RBD (magenta) are shown in cartoon representation. (**B**) The epitope of B38 is shown in surface representation. The contact residues by heavy chain, light chain, or both are colored in cyan, green, and magenta, respectively. The residues on RBD involved in both B38 and hACE2 binding are labeled in red. (**C**) Superimposition of RBD-B38 and RBD-hACE2 [Protein Data Bank (PDB) ID 6LZG]. All molecules are shown in cartoon representation, with the same colors as in (A). hACE2 is colored in light pink. (**D**) The residues involved in hACE2-RBD binding are highlighted in light pink. The residues on RBD involved in both B38 and hACE2 binding are labeled in red. (**E**) The complex structure of SARS-CoV RBD (light blue) and hACE2 (yellow) (PDB ID 2AJF). (**F**) The residues in contact with hACE2 are colored in yellow. The residues are numbered according to SARS-CoV RBD. The residues involved in hACE2 binding of two RBDs are labeled in red. (**G** to **I**) The detailed interactions between COVID-19 virus RBD and CDR loops of the heavy chain. (**J** and **K**) The detailed interactions between COVID-19 virus RBD and CDR loops of the light chain. The residues are shown in stick representation, with the same colors as in (C). The water molecules are shown as red spheres. Single-letter abbreviations for the amino acid residues are as follows: A, Ala; D, Asp; E, Glu; F, Phe; G, Gly; I, Ile; K, Lys; L, Leu; N, Asn; P, Pro; Q, Gln; R, Arg; S, Ser; T, Thr; V, Val; W, Trp; and Y, Tyr.

As is consistent with the binding affinity between RBD and B38 or H4, stable complexes were obtained in both RBD-B38 and RBD-H4 mixtures (fig. S2). The complex crystal structure of RBD-B38 Fab was solved at 1.9-Å resolution (table S2). Three complementarity-determining regions (CDRs) on the heavy chain and two CDRs on the light chain are involved in interaction with RBD (Fig. 4, A, B, and G to K). The buried surface area of heavy and light chains on the epitope is 713.9 and 497.7 Å, respectively. There are 36 residues in the RBD involved in the interaction with B38, in which 21 residues and 15 residues interact with heavy and light chains, respectively (table S3 and Fig. 4B). Sequence alignment indicates that only 15 of the 36 residues in the epitope (defined as residues buried by B38) are conserved between COVID-19 virus and SARS-CoV (Fig. 4, D to F, and fig. S3). Notably, most contacts in the interface between B38 and RBD are hydrophilic interactions (table S4). Water molecules play an important role in the binding between COVID-19 RBD and B38 (Fig. 4, G and I to K). These differences explain the B38-specific binding to the COVID-19 virus rather than SARS-CoV.

To explore the structural basis for B38 blocking the interaction between COVID-19 virus RBD and ACE2, the complex structures of RBD–B38-Fab and RBD-hACE2 were superimposed. Both the V_H_ and V_L_ of B38 would sterically hinder ACE2 binding (Fig. 4C). Notably, the RBDs in B38-bound form and hACE2-bound form have no notable conformational differences, with a Cα root mean square deviation of 0.489 Å (for 194 atoms). Further analysis indicated that 18 of the 21 amino acids on the RBD are involved in binding both B38 and ACE2 (Fig. 4D), which explains why B38 abolishes the binding between COVID-19 virus RBD and the receptor.

As the COVID-19 outbreak continues to spread, characterization of the epitopes on the COVID-19 virus RBD will provide valuable information for vaccine development. Furthermore, the molecular features of the neutralizing antibody targeting epitopes are helpful for the development of small-molecule or peptide drugs and inhibitors. The neutralizing antibodies themselves are also promising candidates for prophylactic and therapeutic treatment against the COVID-19 virus.

## Supplementary Materials

science.sciencemag.org/content/368/6496/1274/suppl/DC1 Materials and Methods Figs. S1 to S3 Tables S1 to S4 References (*19*–*22*) MDAR Reproducibility Checklist

## Appendix

View/request a protocol for this paper from *Bio-protocol*.
